# Reconstituted HDL: Drug Delivery Platform for Overcoming Biological Barriers to Cancer Therapy

**DOI:** 10.3389/fphar.2018.01154

**Published:** 2018-10-15

**Authors:** Sangram Raut, Linda Mooberry, Nirupama Sabnis, Ashwini Garud, Akpedje Serena Dossou, Andras Lacko

**Affiliations:** Lipoprotein Drug Delivery Research Laboratory, Department of Physiology and Anatomy, University of North Texas Health Science Center, Fort Worth, TX, United States

**Keywords:** rHDL, tumor targeting, SR-B1 receptor, cancer therapy and imaging, cholesterol, HDL, biological barriers

## Abstract

Drug delivery to malignant tumors is limited by several factors, including off-target toxicities and suboptimal benefits to cancer patient. Major research efforts have been directed toward developing novel technologies involving nanoparticles (NPs) to overcome these challenges. Major obstacles, however, including, opsonization, transport across cancer cell membranes, multidrug-resistant proteins, and endosomal sequestration of the therapeutic agent continue to limit the efficiency of cancer chemotherapy. Lipoprotein-based drug delivery technology, “nature’s drug delivery system,” while exhibits highly desirable characteristics, it still needs substantial investment from private/government stakeholders to promote its eventual advance to the bedside. Consequently, this review focuses specifically on the synthetic (reconstituted) high-density lipoprotein rHDL NPs, evaluating their potential to overcome specific biological barriers and the challenges of translation toward clinical utilization and commercialization. This highly robust drug transport system provides site-specific, tumor-selective delivery of anti-cancer agents while reducing harmful off-target effects. Utilizing rHDL NPs for anti-cancer therapeutics and tumor imaging revolutionizes the future strategy for the management of a broad range of cancers and other diseases.

## Introduction

Our laboratory has been engaged in reconstituted high-density lipoproteins (rHDL) drug delivery research for more than a decade, focusing on formulating and evaluating rHDL drug delivery vehicles for a multitude of anti-cancer agents. Lipoprotein drug transporters have traditionally been used to deliver hydrophobic and amphiphilic drugs. Recently, our laboratory and others have succeeded in the encapsulation of hydrophilic drugs, including doxorubicin (Yuan et al., 2013). Our hypothesis of utilizing the rHDL drug delivery platform for cancer therapy is based on our understanding of the process of ‘reverse cholesterol transport’ and apolipoprotein/receptor interactions. Endogenous plasma high-density lipoproteins (HDL) delivers cholesterol from peripheral tissues to the liver for metabolism and excretion via a specific receptor, Scavenger Receptor Type B1 (SR-B1) expressed primarily on hepatocytes and steroidogenic tissues (Connelly and Williams, 2004). Several recent articles have shown that SR-B1 is overexpressed by the majority of malignant tumors, promoting their proliferation, and metastasis (Twiddy et al., 2012; Zheng et al., 2013; Yuan et al., 2016; Panchoo and Lacko, 2017). Thus, several laboratories are directing their efforts toward developing rHDL-based formulations to deliver anti-cancer agents to malignant tumors, facilitated by the SR-B1 receptor. While developing/designing a nanoparticulate system for cancer therapy, several biological barriers remain as recently discussed by Blanco et al. (2015). This communication deals with specific biological/physiological challenges based on the innate ability of the rHDL drug delivery platform to overcome them and considering optimization strategies to improve the drug delivery performance of rHDL.

**FIGURE 1:**
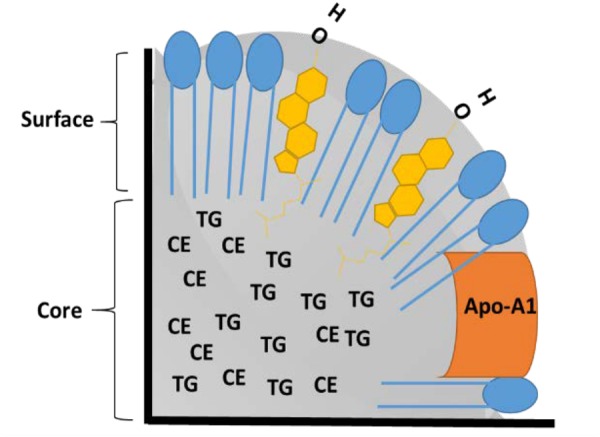
Schematic of cross section of high-density lipoproteins (HDL) nanoparticle (NP). Phospholipids are shown in blue, free cholesterol is shown in yellow, TG: Triglycerides, CE: Cholesterol Esters, and Apo A-1: Apolipoprotein A-1.

The basic structure of rHDL NPs resembles those of their natural counterparts, circulating HDL particles. Details of the structure and composition of lipoproteins have been described earlier (Damiano et al., 2013; Pownall et al., 2016; Thaxton et al., 2016). Briefly, lipoproteins are composed of triglycerides (TG) and cholesterol esters (CE) as core components, phospholipids, and unesterified cholesterol in their outer monolayer, and amphipathic apolipoproteins on their surface that facilitate solubility and stability in the bloodstream (**Figure 1**). Although there are several classes of lipoproteins (VLDL, IDL, LDL, and HDL) in the blood of mammals, the present paper focuses only on high-density lipoproteins because of their preferred use in drug delivery research (Ng et al., 2011; Kuai et al., 2016). Other lipoproteins (VLDL, IDL) and their synthetic analogs have not been studied extensively as drug delivery vehicles although they are known to bind and transport several drugs in blood circulation (Yamamoto et al., 2017). Synthetic LDL nanoparticles (NPs) have been used for drug delivery and diagnostic imaging (Corbin et al., 2006; Corbin and Zheng, 2007).

Apolipoprotein A-1 (Apo A-1) is a major protein component of HDL which also serves as a ligand for the SR-B1 receptor. Several methods have been used to assemble rHDL NPs. including cholate dialysis, sonication, thermal cycling, and microfluidics (Kuai et al., 2016). Synthetic HDL NPs have been prepared using self-assembling peptides (Zhang et al., 2010), polymers (Sanchez-Gaytan et al., 2015), and inorganic template ingredients (McMahon et al., 2017). These platforms have been used for drug delivery, siRNA delivery, and modulating cellular cholesterol levels. The rHDL NPs, Apo A-1 protein, and its mimetic peptides are also known to have anti-inflammatory and anti-apoptotic properties. This feature of potential HDL therapeutics has been reviewed by Barter et al. (2004) and Navab et al. (2007). In this review, we are focusing on the challenges (immune system, hemodynamics, cell membrane transport, and drug resistance) faced by drug delivery systems and how these barriers may be effectively overcome by the rHDL platform for cancer therapy.

## Interaction of rHDL With Blood and Immune Cells

The innate immune system (IIS) is the first line of defense against external pathogens or foreign substances, including NPs. The IIS consists of epithelial barriers, phagocytes, dendritic cells, and plasma proteins (complement). The complement system, one of fundamental components of innate immunity is highly organized and designed to remove/clear ‘foreign’ substances (Medzhitov and Janeway, 2002). The complement system has several protein components that are activated once the pathogen/foreign substances are encountered and subsequently undergoes a cascade of reactions to clear the pathogen/foreign substance(s). There are three branches of the complement system viz classical, lectin and alternative pathway responding to different types of pathogens/foreign sunstances. The classical pathway involves binding of C1q protein to antigen-antibody complex marking it to be cleared. The lectin pathway involves the binding of the carbohydrate component of the lectin to carbohydrates on the surface of pathogens thus initiating the cascade reaction for its removal. An alternative pathway may be activated spontaneously by adsorption of the complement proteins to the surface of pathogens, externally injected materials, including potentially all NP formulations (Moghimi et al., 2011; Szeto and Lavik, 2016). The complement system is activated very rapidly following injury or introduction of external materials into the circulation, therefore, it is critical to evaluate the immune response to NPs immediately following their administration. It is also important to select early time points when evaluating immune responses to novel NP systems as their blood residence times vary markedly, presumably due to the complement response. Other circulating and resident immune cells facilitate clearance and opsonization of pathogens in conjunction with the complement system. Immune cells use a diverse set of receptors (e.g., toll-like receptors, TLRs and C-type lectin receptors, CLRs) to recognize ‘self’ and ‘non-self’ antigens on the pathogen or on the nanomaterial surface. Granulocytes (mostly neutrophils) and antigen presenting cells (APC; monocytes, macrophages, and dendritic cells) phagocytose and degrade pathogens/nanomaterials.

Because the rHDL complex is assembled from essentially the same constituents as endogenous HDL, rHDL also mimics its endogenous counterpart as reflected by its function of delivering drugs to cancer cells via the SR-B1 receptor (Cruz et al., 2013). Exposure of Apo A-1 and lipids to the external environment makes them accessible to innate immune cells and complement proteins. However, because the phospholipids (e.g., phosphatidylcholine) and Apo A-1 (used to construct rHDL NPs) would be recognized as ‘self’ (endogenous blood components) they are not expected to elicit an immune response (Hogquist et al., 2005). To the best of our knowledge, there are no reports of adverse immune responses to rHDL in small animals or humans. Moreover, recent clinical trials (TANGO) conducted by Cerenis Therapeutics testing an engineered HDL mimetic NP formulation (CER-001) in human subjects, did not reveal any adverse reactions, compared to control (Tardif et al., 2014). Several other clinical trials have also demonstrated the safety of rHDL formulations (Nanjee et al., 1999; van Oostrom et al., 2007). The overall maximum tolerated dose (MTD) has been determined to be between 10 and 30 g of synthetic HDL (Kuai et al., 2016). Thus, it is feasible to inject a 500–1500 mg drug dose (assuming 5% loading in rHDL) to patients without anticipating adverse effects from the rHDL NPs (Kuai et al., 2016).

**FIGURE 2:**
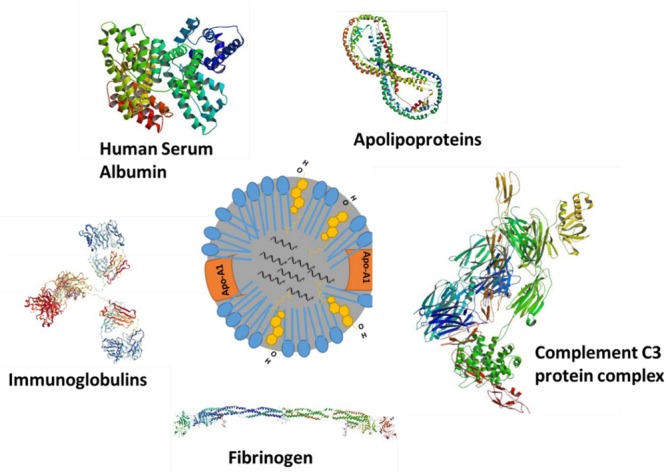
Various types of protein that can adsorb on NPs injected in blood circulation.

Apart from complement activation, several other plasma proteins can adsorb onto the NP surface and modify its surface properties (**Figure 2**). Adsorption or binding of different serum proteins determines the fate of injected NPs. Opsonins such as complement proteins and immunoglobulin adsorption will facilitate nanomaterial removal from the plasma while adsorption of dysopsonins such as bovine serum albumin, apolipoproteins will enhance the circulation time via avoiding the mononuclear phagocyte system. Barrán-Berdón et al. (2013) performed time-evolution studies for investigating the adsorption of different types of proteins to the surface of NPs Their findings indicate that apolipoproteins (Apo A-1, Apo C-II, Apo D, and Apo E), complement component proteins (C1q, C1r, C1s, C3OS, C4-B OS, C5OS, C6OS, C7OS, C8 Alpha Beta and gamma, C9OS, BOS, HOS, and I OS), fibrinogen (alpha, beta, and gamma), and several additional serum proteins, including IgG, serum albumin, serotransferrin, and vitronectin may be associated with the externally introduced NPs (Barrán-Berdón et al., 2013). Lundqvist et al. (2008) showed that polystyrene NPs (PSNP) adsorb to different plasma proteins using mass spectrometry analysis. In general, large 100 nm PSNPs were found to complex with immunoglobulins while smaller 50 nm particles were found to be complexed with apolipoproteins, suggesting that particle size may determine the kind of protein corona acquired by the particles and their role in enhancing the removal from or extended stay in the circulation. The same authors reported that the HDL in plasma binds to copolymer NPs (Hellstrand et al., 2009). These observations have important implications as bound apolipoproteins or entire endogenous HDL complexes may carry NPs to the SR-B1 receptors, expressed by liver cells and macrophages. Several other articles report on the relationship between NP surface properties (size, charge, and functional groups) and protein adsorption (Tenzer et al., 2011; Barrán-Berdón et al., 2013). Unlike rHDL, Endogenous plasma HDL size is known to vary (Lagrost et al., 1996). In-depth studies are needed for drug-loaded synthetic or rHDL NPs to study the serum protein adsorption (if any) and possible re-distribution of the drug payload to other lipoproteins in the plasma. The distribution of payload will also depend upon its hydrophobic characteristics. Findings of McConathy et al. (2011) suggest only a limited re-distribution of a fluorescent lipid analog, compared to the distribution of cholesteryl esters (McConathy et al., 2011).

Among several safety metrics, hemolysis is often investigated to indicate toxicity following administration of a nanoformulation. Hemolysis may involve direct erythrocyte damage and/or an immune-mediated hemolytic process. Generally, positively charged NPs tend to damage erythrocytes in a dose-dependent manner, as found with C60 fullerenes (Bosi et al., 2004), PAMAM (Domański et al., 2004), carbosilane (Bermejo et al., 2007), polypropylene imine (Agashe et al., 2006), and polylysine (Shah et al., 2000) dendrimers. In the case of drug loaded rHDL NPs, several reports indicate no hemolysis when NPs were incubated with red blood cells, perhaps due to the absence of SR-B1 receptor expression by erythrocytes (Oda et al., 2006; Yuan et al., 2013; Lu et al., 2015). Thus, rHDL drug formulations seem to be safe based on their interactions with blood cells and immune components.

Interaction of rHDL with macrophages in atherosclerotic plaques is well documented. HDL interacts with ABCA1 and ABCG1 (ATP binding cassette family) receptors to acquire cholesterol from macrophage foam cells in atherosclerotic plaques, considered to be contributing to its anti-atherosclerotic effect, and attributed to the inverse relationship between HDL-C levels and the risk of coronary heart disease (Yvan-Charvet et al., 2010). Synthetic HDL has been used to image atherosclerotic deposits by delivering radio-imaging agents to macrophages in plaques (Frias et al., 2007; Cormode et al., 2010). Similar to its endogenous counterpart, rHDL can also interact with macrophages and can facilitate cholesterol efflux. However, this interaction may be altered by modifying the Apo A-1 amino acid sequence (without changing its ability to bind to SR-B1) thus providing an important tool to develop novel therapeutic strategies (von Eckardstein et al., 1993). The interaction of rHDL with tumor macrophages may also be exploited to develop an effective immunotherapy strategy (Norata et al., 2012). This interaction may further be exploited to control the expansion of myeloid-derived suppressor cells (MDSC) in infectious diseases, cancer, inflammation and consequently reversing the suppression of the immune response (Plebanek et al., 2018). On the other hand, HDL carrying sphingosine-1 phosphate has been shown to suppress components of the immune system, a potentially valuable tool for treating auto-immune disorders (Blaho et al., 2015). Thus, based on the payload, rHDL can be targeted to activate or suppress specific functions of the immune system.

## Hemorheology and Blood Vessel Fluid Dynamics for rHDL

**FIGURE 3:**
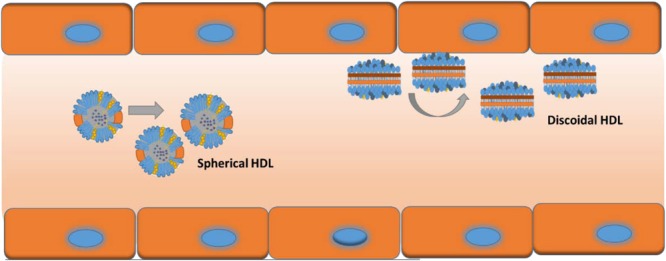
Possible vessel dynamics of spherical (toward center of vessel) vs discoidal (toward vessel wall) HDL NPs in a blood vessel.

Movement of NPs in the bloodstream, adhesion to endothelial cells, and extravasation into the leaky tumor vasculature are highly dependent on the NP geometry. Decuzzi et al have discussed how the different shape/size may impact circulation, margination, adhesion to vessel walls and cellular uptake of the NPs (Decuzzi et al., 2009). Decuzzi et al. (2009) and others have also shown that non-spherical particles are more likely to move closer to the vessel wall due to tumbling and rolling dynamics than spherical ones which tend to stay toward the center of the vessel lumen; parallel to vessel walls (Decuzzi et al., 2005; Shah et al., 2011; Tan et al., 2013; **Figure 3**). Two types of rHDL NPs have been reported in the literature; discoidal; and spherical (Kingwell et al., 2014). However, there are no studies evaluating their vessel dynamics in theoretical models or in animals including the bio-distribution, and intended pharmacodynamics and anti-tumor effects. Moreover, these different shapes of HDL NPs may have different affinities toward SR-B1 receptors, and the ABCA1 and ABCG1 transporters.

Nanoparticle size will also play role in blood circulation residence time. It is known that larger NPs (>200 nm) tend to accumulate in the liver and spleen while smaller ones (<10 nm) tend to get cleared by the kidney (Alexis et al., 2008; He et al., 2010). Thus, size considerations along with adsorption properties will likely, collectively affect the mean blood residence time of NP formulations, including drug loaded rHDL NPs. Moreover, NP surface charge will also contribute to fluid dynamics in the blood circulation. Generally, highly positively charged NPs are easily removed from the blood circulation compared to highly negatively charged NPs (Arvizo et al., 2011). In the case of rHDL NPs, their surface charge may be easily manipulated by using different phospholipid surface components, thus controlling the time in the circulation. The rHDL NPs seem to have an only minimal impact on their overall size when encountering blood components. Skajja et al investigated the stability of iron oxide loaded rHDL NPs *in-vitro* as well as in animals. After incubating the rHDL NPs in plasma for 24 h, the change in size was less than 4% demonstrating the absence of protein adsorption. Moreover, Skajaa et al. (2011) reported that after 24 h incubation NPs remained individually dispersed without aggregation, further at testing to the robust nature of rHDL NPs (Skajaa et al., 2011). The extended circulation times (>24 h) of rHDL formulations have been discussed by elsewhere (Kuai et al., 2016) acknowledging that in clinical studies SRC-rHDL, CSL-111, CSL-112, Pro Apo A-1 liposomes, ETC-216, CER-001, and ETC-642 all displayed extended circulation times with no major safety/toxicity issues. (Kuai et al., 2016).

## Extravasation and rHDL

Numerous research articles have discussed the potential of NPs for targeted drug delivery to cancer cells and tumors. An ideal nanocarrier is expected to have a small size, the extended residence time in the circulation, biocompatibility, and absence of immunogenicity. Although the drug-containing NP formulations exhibited much better delivery efficiency compared to the free drug formulations, they were not as efficacious in limiting off-target effects (Desai, 2012). This discrepancy in clinical translation of nanomedicines has been discussed by several authors (Kamaly et al., 2012; Xue et al., 2014; Min et al., 2015). A crucial element could be a variation of the NP uptake by the reticuloendothelial system in humans compared to that in immune-deficient small animal models. In addition, the physiology, in spontaneously emerging tumors, compared to xenografts may vary significantly. Furthermore, huge dose discrepancies between those administered to humans compared to those used to treat small animals may result in different degrees of drug retention. Heterogeneity in tumor vasculature and the size of the NPs involved may further impact the drug distribution. More importantly, despite an overall increase in the amount of drug delivered to the tumor via NPs, a significant portion of the tumor cells may still have only limited exposure to the drug.

The drug delivery via NPs usually occurs via extravasation of NPs through the leaky tumor vasculature (passive targeting) or targeted delivery via ligand modified surface of the drug carrier (active targeting) (Torchilin, 2010; Ngoune et al., 2016). Although enhanced permeability and retention (EPR) plays an important role in the passive accumulation of nanoformulations at the tumor site, it is dependent on tumor type and the organ in which the malignancy resides (Yokoi et al., 2014b). Studies by Chen et al have demonstrated multi-functional rHDL NP platform for tumor targeting and imaging via both non-specific accumulation and specific binding to angiogenically activated blood vessels (Chen et al., 2010). In this study, the authors decorated rHDL with amphiphilic gadolinium chelates and fluorescent near infra-red (NIR) imaging dye. Angiogenic endothelial cells were targeted via rHDL that was functionalized with αvβ3-integrin-specific RGD peptides (rHDL-RGD). Non-specific RAD peptides were conjugated to rHDL NPs as a control (rHDL-RAD). The *in vitro* studies indicated a clear distinction between non-specific and specific uptake of the two types of NPs. All 3 NPs (rHDL-RGD, rHDL-RAD, and rHDL) were phagocytosed by macrophages, while the endothelial cells were involved in the uptake of only αvβ3-integrin-specific rHDL-RGD NPs. Furthermore, *in-vivo* studies using NIR and MR imaging demonstrated that rHDL-RGD was associated with tumor endothelial cells, whereas HDL and rHDL-RAD NPs were mainly found in the interstitial space. Thus, it is possible to re-route the rHDL NPs via active targeting.

In another study, Zhang et al. (2010) have demonstrated that sub -30 nm HDL mimicking NPs, functionalized with an EGFR targeting ligand, preferentially accumulated in the tumor interstitial spaces, suggesting that the small size, neutral surface charge, specific targeting ligand and long circulation half-life were instrumental in successful extravasation of the NPs into the tumor mass (Zhang et al., 2010). Thus, rHDL possessed therapeutic potential and versatility in mediating Chol-siRNA-VEGF direct cytosolic delivery for target-specific anti-angiogenic therapy in breast cancer. Another factor involved in the successful extravasation of nano-formulations is the collagen content in the capillary walls in the tumor vasculature (Yokoi et al., 2014a) A similar study may reveal whether the capillary collagen content has any effect on extravasation of rHDL NPs.

The potential utility of HDL mimicking NPs as programmable, and biocompatible drug delivery vehicles suitable for the targeted delivery of tumor imaging and anti-cancer agents has now been established. The HDL NPs owing to their small size, close to neutral charge and long circulation time are able to move out of the circulation and accumulate in malignant tissues. Aforementioned studies also demonstrate the ability to reroute rHDL from its natural target to tumor blood vessels and its potential for multimodal imaging of tumor-associated processes. An analysis of NP payload uptake and drug delivery on the microscopic level in small animal studies will be essential to understanding the efficiency of therapeutic effects. Based on clinical studies, rHDL has already been proven safe for human administration (Krause and Remaley, 2013).

## Cellular Membrane Transport and Endosomal Escape of rHDL

Unlike small molecules which readily diffuse into cells and tissues, drugs, encapsulated in NPs must have facilitated delivery across cell membranes. Cellular internalization and endosomal/lysosomal entrapment are of great concern in NP design. Whether trapped in compartments or subjected to the low pH and enzymatic environment of the lysosome, endosomal escape is necessary for the efficient delivery of the NP drug cargo to the tumor site. Size, charge and surface decoration of NPs determine which endocytic pathway they may enter and their subsequent intracellular fate (Blanco et al., 2015). Concerns about the endosomal escape of therapeutic agents became paramount with the advent of RNA interference for clinical applications. However, nucleic acids, charged molecules with high molecular weight (5–1000s kD), are easily degraded by endogenous nucleases and are thus unable to reach their pharmaceutical target without assistance. To achieve an effective cellular response, nucleic acid therapeutics has to involve an endosomal escape process.

Several pathways exist for movement of the NP or its cargo into the cell interior. Endocytosis is a process of forming vesicles and moving them toward intracellular targets usually involving clathrin-coated pits where the specific receptors reside (e.g., uptake of low density lipoprotein (LDL) and the LDL receptor described by Brown and Goldstein (Doherty and McMahon, 2009; Goldstein and Brown, 2009; Kaksonen and Roux, 2018). Several clathrin-independent pathways such as endophilin-mediated, flotillin-mediated and macropinocytosis have also been reported (Doherty and McMahon, 2009; Amaddii et al., 2012; Sorkina et al., 2013; Ferreira and Boucrot, 2017). Cargo contained in endosomes can be recycled back to the plasma membrane or other organelles, delivered to the lysosome for degradation, or undergo a process called transcytosis (Elkin et al., 2016). Once vesicles pinch off from the plasma membrane, loaded with cargo, they mature into early endosomes and are sorted to determine their final destination (Salzman and Maxfield, 1988; Huotari and Helenius, 2011). As endosomes continue to mature from the early to the late stage and into the lysosome, they become progressively more acidic due to the ATP-dependent pumping of hydrogen ions into the lumen (Mellman et al., 1986). The early endosomal interior has a pH in the range of ≈6.5 compared to ≈5.5 for late endosomes. These pH changes are needed for the dissociation of receptors from their ligands in order to be processed via recycling of the receptor or ligand degradation. Degradation of the cargo occurs when the late endosome fuses with the lysosome, creating an endolysosome with a pH of ≈4.5 exposing the cargo to a full spectrum of hydrolases (Huotari and Helenius, 2011). The endolysosomal environment is considered detrimental to targeted NP cargo delivery, particularly to intracellular nucleic acid transport (Blanco et al., 2015).

### Strategies for Endosomal Escape

Strategies for the endosomal escape is a wide-ranging topic that merits a review of its own. However, these strategies could represent key steps in the design, and manufacturing of NP formulations. Rather than designing and preparing complex formulations, direct cytosolic delivery of the therapeutic payload could be achieved with a drug delivery NP platform such as the reconstituted/recombinant high-density lipoproteins (rHDL). The receptor for HDL, Scavenger Receptor Class B, Type 1 (SR-B1) facilitates the endogenous delivery mechanism directly into the cytosol, a process known as selective lipid uptake (Zhang et al., 2009)

#### SR-B1 and Selective Uptake

There are five isoforms of SR-B1 from splice variants encoded by the SCARB1 gene. The first isoform was named as SR-B1 and shown to be the HDL receptor (Acton et al., 1996; Calvo et al., 1997). The receptor protein consists of 509 amino acids and has an apparent molecular weight of 82–85 kDa. Isoform 3 has a length of 552 amino acids and has been set as the canonical sequence (Gutierrez-Pajares et al., 2016). The highest expression of SR-B1 in normal tissues can be found in the liver and steroidogenic organs (Landschulz et al., 1996; Calvo et al., 1997; Arenas et al., 2004; Shahzad et al., 2011). In murine adrenocortical cells and an SR-B1 transfected Chinese hamster ovary cell line, SR-B1 appears to co-localize with caveolae (Babitt et al., 1997). In hepatocytes, SR-B1 interacts with an adaptor protein, PDZK1, which mediates its localization and function (Kocher and Krieger, 2009). When rat SR-B1 was over-expressed in an adrenal cell model, the receptor was reported to dimerize, restructure plasma membrane architecture, and to increase cholesteryl ester uptake (Reaven et al., 2006). Malignant tissues have been found to over-express the SR-BI receptor (Lacko et al., 2002, Shahzad et al., 2011) and patients with high expression levels have a tendency for worse prognoses (Shahzad et al., 2011; Schörghofer et al., 2015; Yuan et al., 2016; Feng et al., 2018). It is not known if over-expressed SR-B1 is in a dimeric or oligomeric state in cancer cells, but it is known that cancer patients have lowered blood cholesterol levels, particularly HDL cholesterol (Rose et al., 1974; Fiorenza et al., 2000; Shah et al., 2008; Muntoni et al., 2009).

#### HDL and Small Molecules

**FIGURE 4:**
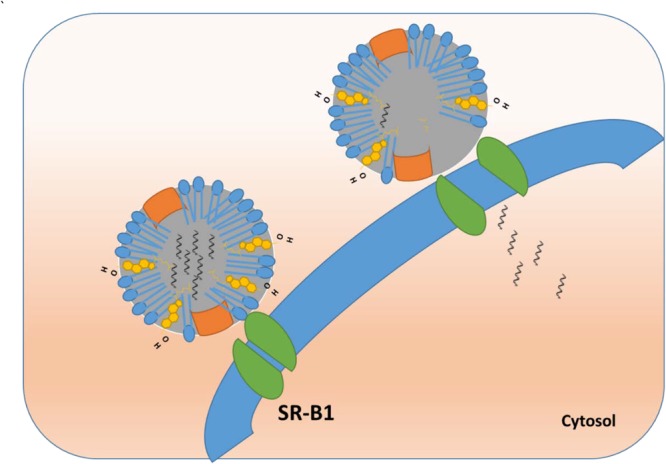
Endosomal escape and direct cytosolic delivery of cargo using rHDL NPs.

In case of HDL, the ‘selective lipid uptake mechanism’ involves HDL docking with its receptor (Murao et al., 1997) followed by internalization of only the core contents (cholesteryl esters), not the whole particle (**Figure 4**). The exact mechanism of how SR-B1 achieves selective lipid uptake is yet to be fully elucidated. It was postulated that SR-B1 forms a hydrophobic channel or pore when docked with HDL to allow passage of cholesteryl esters across the cell membrane (Rodrigueza et al., 1999). It has been reported that HDL undergoes retroendocytosis, in which the lipoprotein is taken up just inside the plasma membrane, delivers its cargo, and exits the cell without entering the endocytic pathway (Pagler et al., 2006; Sun et al., 2006; Rohrer et al., 2009). It is possible that the formation of an SR-B1 pore allows all hydrophobic materials (other than cholesteryl esters) to traverse the cell membrane, particularly when the receptor is over-expressed in cancer (Mooberry et al., 2010).

One of the early reports in the literature observed that 82% of paclitaxel from the core of rHDL was taken up by the cell in a selective SR-B1-like mechanism (Mooberry et al., 2010). The drug uptake could be partially blocked by Apo A-I, discoidal Apo A-I/PC complexes or isolated human HDL (Mooberry et al., 2010). Further validation of selective delivery was shown with a model compound dilauryl fluorescein taken up in SR-B1-transfected cells that could be limited by adding either Apo-AI or excess rHDL (McConathy et al., 2011). Yang et al have also shown that gold templated HDL mimetic NPSs were found in the cytoplasm after administration to mice (Yang et al., 2013). Selective cytosolic delivery was also shown with an Apo A-I mimetic peptide/phospholipid nanocarrier created to carry the hydrophobic fluorophore, DiR-BOA (1, 1′-dioctadecyl-3, 3, 3′, 3′-tetramethylindotricarbocyanine iodide bis-oleate) in its core (Zhang et al., 2009; Case et al., 2012). Uptake of DiR-BOA did not co-localize with the lysosomal marker, LysoTracker. Z stacks obtained by confocal microscopy showed a cytosolic location for the fluorophore (Zhang et al., 2009). Additional reports with other drugs, such as valrubicin and fenretinide, have shown that SR-B1-mediated drug uptake from rHDL can be inhibited via the Block Lipid Transporter 1 (BLT1) or antibody blocking of SR-B1 (Sabnis et al., 2012, 2013; Johnson et al., 2017).

In an elegantly designed study, Lin et al used a multi-fluorophore-labeled NP, termed HDL-mimicking peptide phospholipid scaffold (HPPS) to probe uptake via the SR-B1-mediated pathway (Lin et al., 2014). The core of the HPPS NPs was fluorescently labeled with either DiR-BOA or Fluo-BOA (dioleyl fluorescein) (Zhang et al., 2010). Rhodamine-B-labeled phospholipids were incorporated into the phospholipid monolayer of the HPPS NPs. An 18-amino acid Apo A-I mimetic peptide was conjugated to a FITC label and complexed with HPPS NPs. The selective uptake was monitored in SR-B1-transfected cells. Lin et al. (2014) reported that a synthetic cholesteryl ester analog selectively entered the cells, leaving the phospholipids and peptide on the exterior of the cell. (Lin et al., 2014). Cytosolic delivery of the hydrophobic core content of the HPPS NP could not be inhibited by temperature changes or energy depletion. Utilizing endocytosis inhibitors to target either clathrin or actin revealed that the SR-B1-mediated uptake was an endocytic process. In contrast, the cytosolic delivery could be affected by disruption of lipid rafts or caveolae or the SR-B1 inhibitor, BLT1. However, it is known that disruption of lipid rafts/caveolae has minimal effect on the uptake of CE from HDL (Rhainds et al., 2004; Wustner et al., 2004). Mooberry et al. (2010) have shown that only 18% of the paclitaxel-loaded rHDL entered the cell via whole particle uptake, suggesting that these particles were endocytosed (Mooberry et al., 2010). The quantitative aspects of the multiple mechanisms involved in the uptake of the drug payload from rHDL NPs are yet to be elucidated.

#### HDL and Nucleic Acids

It important to cite the pioneering work done by several researchers in siRNA delivery and SR-B1 involvement. Bijsterbosch et al studied the biodistribution and organ accumulation of phosphorothioate antisense oligo whereby most of it (>90%) was found to be in the liver at 90 min indicating that scavenger receptors were involved (Bijsterbosch et al., 1997, 2000). Regarding the selective delivery of nucleic acids, the molecular weight differences are striking; the molecular weight of an average siRNA is approximately 20-fold that of the average drug molecule. However, it appears that direct cytosolic delivery of nucleic acids also occurs through SR-B1. It has been reported that endogenous HDL in the circulation carried endogenous microRNA and delivered it to target cells through SR-B1 (Vickers et al., 2011). Uptake of a fluorescently labeled siRNA via rHDL in SR-B1-expressing tumors and the liver in an ovarian cancer mouse model illustrates the ability of rHDL to deliver nucleic acids through SR-B1 (Shahzad et al., 2011). Using a fluorescently labeled cholesterol-conjugated siRNA (FAM-Chol-siRNA) packaged in rHDL, Ding et al observed delivery, specifically to the cytosol in the hepatocellular carcinoma cell line, HepG2 (Ding et al., 2012). Further studies validated the SR-B1-mediated direct cytosolic delivery of the FAM-Chol-siRNA by blocking uptake with endogenous HDL in the MCF7 breast cancer cell line. Secondly, the intracellular location of the FAM-Chol-siRNA was compared to the location of a LysoTracker signal and found to not colocalize (Ding et al., 2014). Other studies used fluorescently labeled transferrin as an endocytosis marker and found no co-localization with siRNA, delivered via an HDL-mimicking NP (Yang et al., 2011).

Direct cytosolic delivery of siRNA could be the explanation as to why HDL-type NPs exhibit favorable *in vivo* efficacy in tumor models (Shahzad et al., 2011; Ding et al., 2012, 2014). Concerns about off-target toxicity toward other SR-B1-expressing organs (particularly the liver) can be allayed by a study in which 72 h after administration, the fluorescent signal was still strong in the tumor, but cleared from the liver (Ding et al., 2014). Furthermore, liver function tests revealed no difference between control groups and rHDL/siRNA therapy groups (Shahzad et al., 2011). Additional preclinical and translational research will be necessary to demonstrate the advantages of an HDL-like NP platform. (McMahon and Thaxton, 2014). An excellent review by McMahon et al. (2016) further discusses HDL and its ability to transport and deliver different RNAi species for therapeutic applications (McMahon et al., 2016).

## Drug Resistance and rHDL

Resistance to therapy (chemo-resistance) leads to relapse and metastasis and thus substantially diminishes the prospects for remission or cure for cancer patients (Zheng, 2017). Consequently, resistance to anti-cancer drugs has continued to be a major impediment to effectively treating many cancers (Robey et al., 2018). The process is known as “multidrug resistance”, is a particularly difficult barrier to overcome, especially during the treatment of metastatic disease (Robey et al., 2018). Consequently, a reversal (Angelini et al., 2008) or prevention (Cole, 2014) of drug resistance, displayed by aggressively growing malignant tumors. Although numerous approaches (Harker et al., 1986; Hyafil et al., 1993; Choi et al., 2010), including administration of drugs (Koo et al., 2008) have been explored, the clinical efficacy of these treatments met only with limited success. Drug resistance thus remains to be a major obstacle in oncology (Robey et al., 2018).

Because the well-described mechanisms of drug resistance (Lin et al., 2014; Robey et al., 2018) often involve pumping units located in the membranes of malignant cells, the idea that *cytoplasmic delivery* of cytotoxic anti-cancer agents could overcome these obstacles has been considered by several investigators (Lin et al., 2014; Yang et al., 2014; Yin et al., 2015). These references include one of the earliest reports, providing data on cytoplasmic delivery of drug payloads via rHDL NPs (Lin et al., 2014). Our laboratory was the first to report that paclitaxel delivery to prostate cancer cells takes place primarily via facilitation of the SR-B1 receptor (Mooberry et al., 2010). This delivery route (not requiring the entry of the whole NP into the cell) is known to result in the transport of the lipoprotein payload directly to the cytosol (Ding et al., 2014). Drug resistance to chemotherapy may be avoided by utilizing the SR-B1-HDL interaction. The Thaxton lab has been using the gold templated HDL mimetic NPs to alter the cholesterol flux in cancer (leukemia and lymphoma) cells and to induce apoptosis, thereby completely avoiding the need for the use of cytotoxic drugs (Plebanek et al., 2015; Rink et al., 2017). Because each of these cell types has differing needs for cholesterol, it may be explored to determine whether other drug-resistant solid tumors may be successfully treated using gold core HDL mimetic NPs.

While this mechanism is attractive and our laboratory has now collected additional evidence for the selective delivery of the rHDL drug payload and for the endosomal escape, so far, there is no direct validation available for the hypothesis that rHDL transported therapeutic agents would be able to mediate or limit drug resistance. All indications point to the success of this approach that is likely to have ‘game-changing’ significance in the therapeutics of aggressive tumors and the management of other diseases as well.

## Conclusion

In summary, rHDL NPs are inherently capable of overcoming several biological barriers to cancer therapy. Their small size, intrinsic targeting ability, endosomal escape, demonstrated safety in animal and human studies makes this platform highly attractive for new as well as traditional chemotherapy drugs which suffer from off-target toxicity issues. Liver toxicity could be a potential concern, however, we have not come across any report showing liver toxicity of rHDL delivered drugs in murine models. Our laboratory did not find any change in liver enzymes with STAT3 targeted siRNA for ovarian cancer therapy (Shahzad et al., 2011). Clearly, more research work is needed in this area to establish the effect of various drugs via this delivery system that they may have on hepatocytes and the liver itself. Although several aspects such as drug resistance need to be investigated further, we submit that rHDL based chemotherapy approach will likely shift the risk/benefit ratio associated with current chemotherapy.

## Author Contributions

SR conceived the idea, wrote and coordinated all the sections in the manuscript. LM, NS, AG, AD, and AL have contributed to writing different sections and revising this manuscript.

## Conflict of Interest Statement

The authors declare that the research was conducted in the absence of any commercial or financial relationships that could be construed as a potential conflict of interest.
